# Self-Reported Non-Celiac Wheat Sensitivity in High School Students: Demographic and Clinical Characteristics

**DOI:** 10.3390/nu9070771

**Published:** 2017-07-19

**Authors:** Antonio Carroccio, Ornella Giambalvo, Francesco La Blasca, Rosario Iacobucci, Alberto D’Alcamo, Pasquale Mansueto

**Affiliations:** 1 Internal Medicine, Dipartimento di Biologia e Medicina Interna e Specialistica, University of Palermo, 90133 Palermo, Italy; 2 Internal Medicine Department, Giovanni Paolo II Hospital, 92019 Sciacca, Italy; 3 Department of Economics, Management and Statistics, University of Palermo, 90127 Palermo, Italy; ornella.giambalvo@unipa.it; 4 Dipartimento di Biologia e Medicina Interna e Specialistica, Division of Internal Medicine, Policlinico University Hospital, 90127 Palermo, Italy; francescolablasca@gmail.com (F.L.B.); iacobuccirosario@gmail.com (R.I.); adalcamo@hotmail.it (A.D.); pasquale.mansueto@unipa.it (P.M.)

**Keywords:** self-reported non-celiac wheat sensitivity, non-celiac gluten-sensitivity, epidemiology, prevalence, teenagers, IBS, food allergy

## Abstract

Background: Non-Celiac Wheat Sensitivity (NCWS) has recently been included among the gluten-related disorders. As no biomarkers of this disease exist, its frequency has been estimated based on self-reported symptoms, but to date no data are available about self-reported NCWS in teenagers. Aim: To explore the prevalence of self-reported NCWS in a group of high school students and to study their demographic and clinical characteristics. Methods: The study was performed between April 2015 and January 2016 in two high schools of a coastal town in the south of Sicily (Italy). A total of 555 students (mean age 17 years, 191 male, 364 female) completed a modified validated questionnaire for self-reported NCWS. The subjects who self-reported NCWS were then compared with all the others. Results: Seven individuals (1.26%) had an established diagnosis of CD. The prevalence of self-reported NCWS was 12.2%, and 2.9% were following a gluten-free diet (GFD). Only 15 out of 68 (23%) NCWS self-reporters had consulted a doctor for this problem and only nine (14%) had undergone serological tests for celiac disease. The NCWS self-reporters very often had IBS symptoms (44%). Conclusions: Self-reported NCWS was found to be common in teenagers, with a frequency of 12.2%; the frequency of GFD use was 2.9%, which was much higher than the percentage of known CD in the same population (1.26%). A greater awareness of the possible implications on the part of the subjects involved, and a more thorough medical approach to the study of self-reported wheat-induced symptoms are required.

## 1. Introduction

Non-Celiac Wheat Sensitivity (NCWS) was identified as a distinct clinical condition about 40 years ago [1], but it has only recently been included among the gluten-related disorders. It has been defined as a syndrome due to wheat/gluten ingestion which afflicts patients not suffering from celiac disease (CD) and/or wheat allergy [2]. The NCWS diagnosis is often suggested by the patients themselves, who report gastrointestinal and extra-intestinal symptoms after wheat ingestion and become asymptomatic on a gluten-free diet [3].

However, the lack of diagnostic biomarkers for NCWS is an unsolved problem; consequently, the real frequency and the existence of NCWS itself is debated [4].

Previous works have reported a wide range of self-reported NCWS prevalence, between 0.6% and 13% [5,6,7,8,9,10,11], with lower percentages of adherence to a gluten-free diet (GFD). However, all these previous studies, but one [5], included only adult subjects and none included teenagers.

In our study, we explored the prevalence of self-reported NCWS in a large population of high school students attending two different schools in Sciacca, a middle-sized town in Sicily. We focused on the demographic and clinical aspects of the self-reported NCWS students, comparing them with all the others involved, to search for potential predictors and causal factors of this condition.

## 2. Materials and Methods 

The study was performed between April 2015 and January 2016, in two high schools in Sciacca, a coastal town of 40,000 inhabitants in the south of Sicily (Italy). The two schools were a secondary school focusing on the humanities (“Liceo Classico”) and an arts high school (“Liceo Artistico”). Students were asked to participate in a survey by the Department of Internal Medicine (D.Bi.M.I.S.) of the University of Palermo (Italy), and those who accepted were convened, in groups of about 100, to meetings with the investigators. Each of the five meetings was introduced with a slide presentation by the first author (A.C.), who briefly and simply informed the students about the functional gastrointestinal disorders (i.e., what is “irritable bowel syndrome”, what is “functional dyspepsia”) and the food-related diseases (i.e., what is “celiac disease”, what is “food allergy”). Participants then anonymously completed a modified version of a previously validated questionnaire [7], and the authors (A.C., P.M., R.I., F.L.B.) assisted them when necessary.

### 2.1. Questionnaire

The questions covered general demographic information, including age, sex, parents’ education, and employment categories. A second section covered the presence of symptoms consistent with irritable bowel syndrome (IBS), which was defined in accordance with the Rome III criteria [12], and the participants’ medical history, with particular attention to the occurrence of other gastrointestinal and autoimmune diseases, psychiatric and neurological disorders, and allergic conditions. A third section covered the presence of symptoms related to wheat ingestion: their type, frequency, latency period after eating, and duration; consultations and examinations performed were also recorded, as well as any consequent changes in dietary habits. We considered as NCWS self-reporters the subjects who reported symptoms after wheat ingestion (and symptom disappearance on a wheat-free diet), with a frequency of at least once per week or more; subjects reporting a lower frequency per week were not considered as self-reported NCWS. All the subjects who did not report NCWS or CD were considered controls.

### 2.2. Statistical Analysis

Descriptive statistics, including total numbers, percentages, odds ratio (OR), and relative risk (RR) were performed. The chi-square test was used to compare the frequencies. Parametric variables were expressed as mean and standard deviation (SD), and the differences between the groups analyzed using Student’s *t*-test. A *p*-value of less than 0.05 was considered statistically significant.

Statistical analysis was performed using SAS^®^ 9.4. (SAS Institute Inc., Cary, NC, USA).

The study was registered at clinicaltrials.gov (registration number: NCT03021148).

Exploratory analyses were conducted by fitting a logit model and using multiple correspondence analysis (MCA). MCA allows the analysis of the pattern of relationships of several categorical dependent variables. As such, it can also be seen as a generalization of principal component analysis when the variables to be analyzed are categorical instead of quantitative. The inertia (i.e., the explained variance) of the solution indicates the goodness of fit of the representation, which means how well it fits a set of observations. In our case, where supplementary variables were not used, the model captured 69% of total variance. The interpretation in MCA is based on proximity between points in a low-dimensional map (i.e., two or three dimensions). Specifically, when two or more points are close to each other they appear together in the observations and are similar [13]. To analyze the proximity, an interpretation of the axes is required. Results of MCA suggest interpreting the profiles according to two axes, following parental social status (axis 1) and the gravity of symptoms (axis 2). The results were used for the interpretation of the phenomenon and for comments.

## 3. Results

A total of 555 of the 558 students invited to participate completed the questionnaire; 386 (69%) of them attended the humanistic studies school and 169 the arts school. Seven individuals (1.26%) had an established diagnosis of CD and were excluded from further analysis. Sixty-eight subjects (12.2%) self-reported that they suffered from NCWS; they were 46 females (68%), but no difference was observed between the demographic characteristics of these subjects and those of all the other participants (Table 1).

Only the percentage of the engaged students was slightly higher in NCWS than in controls and tended to be statistically significant (*p* = 0.07). Regarding the clinical manifestations, 30 of the 68 (44%) subjects with self-reported NCWS recorded symptoms which led to an IBS diagnosis, compared with 125 of the 480 control subjects (25%) (chi-square = 10.3057; *p* = 0.0013); the odds ratio for self-reported NCWS subjects when IBS was considered was 2.3 (95% C.I. 1.4–3.9). The relative risk for self-reported NCWS was 2.05 (95% C.I. 1.3–3.2).

**Figure 1:**
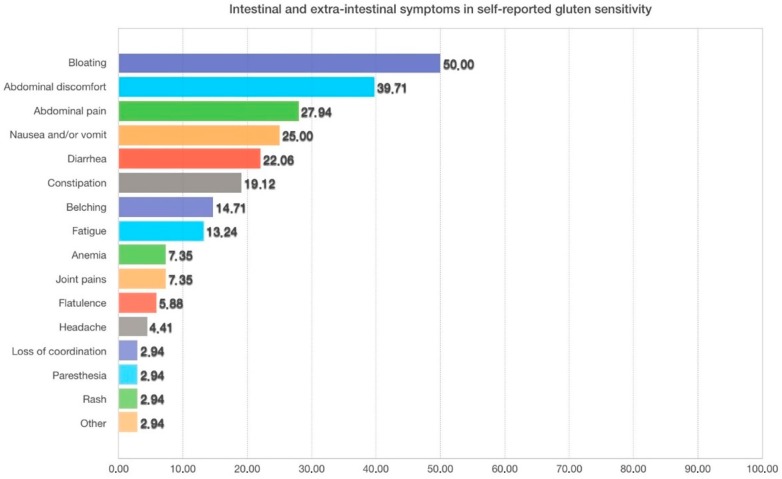
Symptoms caused by wheat ingestion in self-reported NCWS students (the percentage of subjects suffering from each symptom is shown). Multiple symptoms were reported in several subjects.

Figure 1 shows the symptoms caused by wheat ingestion in self-reported NCWS students. Multiple symptoms were recorded; the most frequent gastrointestinal symptoms being bloating (50%), abdominal pain (29%), and nausea (25%); among the extra-intestinal symptoms, the most frequent were joint pain and anemia, reported with a frequency of about 8%.

Self-reported NCWS subjects had been suffering from this condition for a median duration of 12 months (range 2–180 months). Twenty-six percent of these subjects had symptoms regularly, i.e., every time they ate wheat. All the others, in accordance with the inclusion criteria, reported having symptoms at least once per week or more.

**Figure 2:**
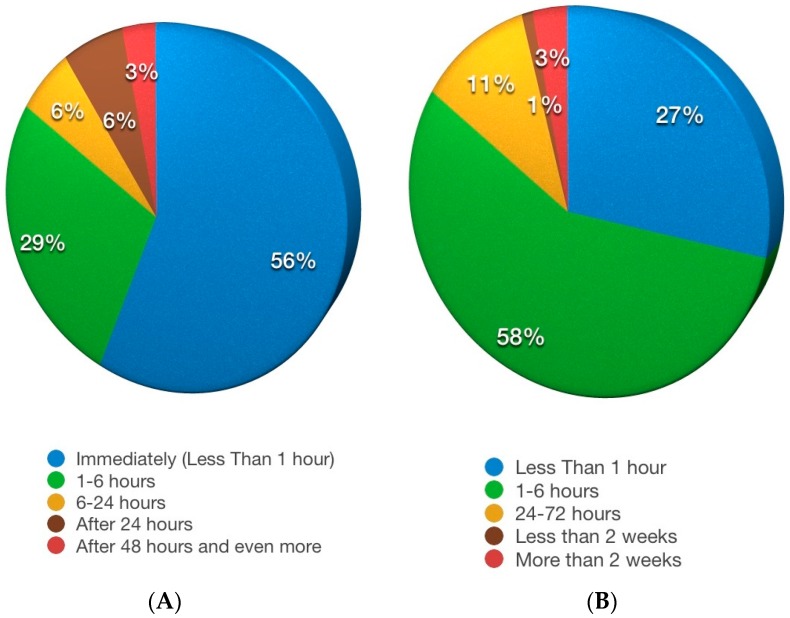
(**A**) Time lapse between wheat ingestion and symptom appearance in self-reported NCWS subjects; (**B**) Duration of the symptoms after wheat ingestion in subjects with self-reported NCWS.

Figure 2A shows the time lapse between wheat ingestion and symptom appearance: most of the subjects reported that the symptoms were almost immediate. About half of the subjects reported symptom resolution within six hours (Figure 2B).

Not all wheat-based foods triggered symptoms to the same extent; pasta and pizza were considered the most troublesome by 47% and 42% of the subjects, respectively, whereas biscuits were considered harmful by 8% of them.

Table 2 shows the diseases which NCWS subjects and controls reported they suffered from. We had stressed that they should report only the diagnoses confirmed by their physicians.

Anxiety, reported by more than half of the subjects in both groups, was the most frequent symptom; both anxiety and depression showed a tendency to be more frequent in self-reported NCWS than in controls, although without a statistically significant difference. The subjects with self-reported NCWS showed a statistically higher frequency of chronic fatigue syndrome, food allergy, and gastro-esophageal reflux disease.

**Figure 3:**
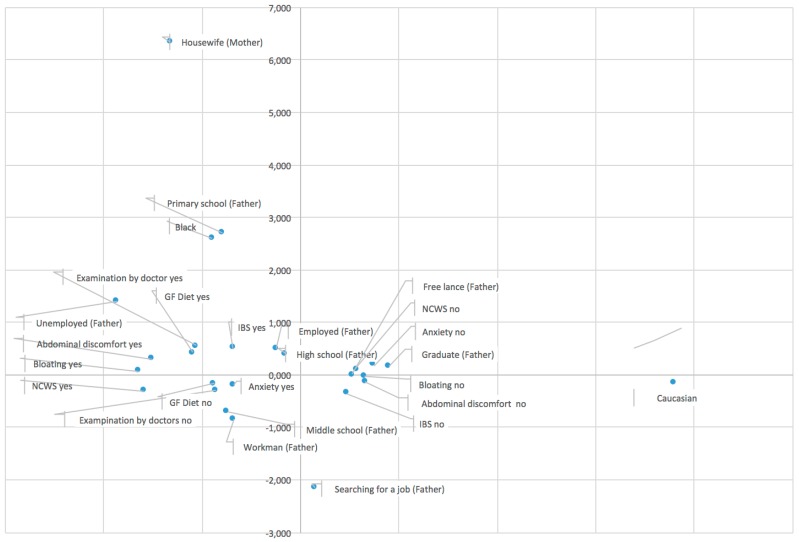
Multiple correspondence analysis shows the relationship between some variables evaluated by the questionnaire including our variable of interest (self-reported NCWS). It identifies some similar groups of individuals following the parental social status (axis 1) and the gravity of symptoms (axis 2).

Multiple correspondence analysis identified some similar groups of individuals, following the parental social status (axis 1) and the gravity of symptoms (axis 2); an association between self-reported NCWS and anxiety was also observed (Figure 3).

Only 15 out of 68 (23%) self-reported NCWS subjects had sought medical advice about this problem and only six of them had consulted a gastroenterologist. Nine (14%) and eight (12%) of them had undergone serologic tests for celiac disease or IgE-mediated wheat allergy, respectively. In all these cases both celiac disease and IgE-mediated food allergy diagnoses had been excluded. Three subjects had undergone upper endoscopy.

Sixteen of the sixty-eight (24%) self-reported NCWS subjects had followed a gluten-free diet (GFD) and fourteen of them (88%) became asymptomatic. In the whole sample population, the percentage of subjects who had undergone a GFD was 2.9% (16 out of 548). However, at the time of this survey, only six subjects were still on a GFD.

## 4. Discussion

Although NCWS is not a well-defined clinical condition as yet, it is characterized by intestinal and extra-intestinal symptoms caused by the ingestion of wheat (and often other cereals), in patients in whom both celiac disease and IgE-mediated wheat allergy have been excluded [2]. The lack of diagnostic biomarkers makes diagnosis difficult and it is based on the clinical benefit of gluten/wheat elimination from the diet and the subsequent response to the double-blind placebo-controlled challenge procedure [14,15]. This is a cumbersome and time-consuming method, used almost exclusively in the research setting.

Consequently, the real frequency of NCWS is unknown. The few previous studies which estimated self-reported NCWS frequency showed a wide range of between 0.6% and 13% [5,6,7,8,9,10,11], but no data have previously been collected among teenagers.

In our study, performed on a group of boys and girls aged between 14 and 19 years, the frequency of self-reported NCWS was 12.2%; this is extremely close to the 13% value reported in 1002 adults in the UK [7]. Interestingly, we used a revised version of the same questionnaire used for the UK study. However, a third study using a similar questionnaire recently reported a lower frequency in the Netherlands: 6.2% [11].

The percentage of our study subjects who had followed a gluten-free diet was 2.9% (16 out of 548), slightly lower than the 3.7% reported in the UK study [7]. The strong similarity between the percentages of self-reported NCWS and self-prescribed wheat-free diet observed in our teenagers and in the adults studied in the UK [6] seems to suggest that self-reported NCWS is a problem perceived in all age groups. Even though most of these subjects (14 out of 16) stated their symptoms improved or were cured on a GFD, we found that the majority were not strictly adhering to an elimination diet at the time of our survey. This result seems to be in contrast with the recent evidence found by our group that 74% of 200 (adult) patients remained on a strict wheat-free diet for a median time of >8 years after NCWS diagnosis [16]. However, it must be emphasized that the cohort of patients included in that previous follow-up study had been officially diagnosed with NCWS after a strict and rigorous protocol, including the double-blind challenge procedure, the best diagnostic means currently available. In contrast, the subjects included in the present epidemiological study had never received a “formal” diagnosis of NCWS. This probably contributed to their decision to abandon the GFD, but does not necessarily mean that they did not benefit from it. Furthermore, the economic cost of the GFD could also have contributed to their abandoning the diet.

On the other hand, the awareness of the subjects of the importance of the gluten-related symptoms observed in our study group and the consequent medical approach were clearly insufficient. Less than one quarter (15 out of 68) of the self-reported NCWS subjects had consulted a physician for this problem, and only nine of them had undergone serologic tests for celiac disease. This underlines that the diagnostic approach to gluten-related diseases needs to be improved in our region, although it does also seem to be a problem in other regions, as highlighted in other papers [17].

Regarding the clinical characteristics of the self-reported NCWS subjects, our study confirmed a strong relationship with IBS symptoms [3,18]: about half of the subjects reported symptoms which led to an IBS diagnosis. The risk of self-reporting NCWS when IBS symptoms are present is two times higher than when they are absent (RR = 2.052), and for individuals with IBS symptoms, the probability of NCWS is more than twice that of individuals without IBS symptoms (ODD ratio = 2.3).

Some of the other clinical characteristics reported (the rapid onset of symptoms after wheat ingestion in more than fifty percent of the subjects, the higher frequency of associated “formal” diagnoses of food allergy) seem to indicate a possible pathogenesis based on a non-IgE-mediated mechanism, as suggested by other studies [19,20]. However, a strong association between food intolerance and IBS, without a clear immunologic mechanism, including a role for fermentable oligo-, di-, mono-saccharide, and polyol (FODMAP) intolerance, is increasingly being demonstrated in the literature [21,22]. Furthermore, in the self-reported NCWS subjects we observed a higher frequency of anxiety and depression than in controls, which was very close to being statistically significant. A similar association (anxiety and self-reported NCWS) was shown by the multiple correspondence analysis.

On the whole, these data seem to confirm that self-reported NCWS subjects could include patients with very different kinds of pathogenesis, including both “organic-immunologic” conditions and psychosomatic disorders. In other words, our epidemiological study uncovered a “pot-pourri” that a double-blind placebo-controlled wheat challenge should help to classify [23].

The limitations of our study must be underlined: above all, the data are based on self-reporting. Consequently, symptoms, clinical course, and concomitant diagnoses and medical conditions were recorded exclusively on the basis of participant accounts. However, we attempted to reduce possible misunderstanding by means of a short educational talk which preceded the questionnaire. This choice itself, however, could have somewhat affected the suggestibility of the participants and we cannot rule out that it may have led to an over-reporting of the food-related troubles. Furthermore, it is important to underline that the most frequent symptoms reported (bloating and abdominal pain) could suggest an intolerance to fermentable oligosaccharides, disaccharides, monosaccharides, and polyols (FODMAP intolerance). The hypothesis that FODMAP intolerance may play a role in the pathogenesis of the symptoms reported could also be suggested by the finding that pasta and pizza were the most troublesome foods, and that onion or garlic, two other foods with a high FODMAP content, are very often included in their toppings/sauces. Obviously, in any case, we must stress that no clinical or pathogenic implications can be suggested by an epidemiological study such as ours. Finally, our definition of self-reported NCWS (“symptoms after wheat ingestion with a frequency of at least one time a week or more”) could be considered not sufficiently appropriate.

In conclusion, we showed that NCWS is quite common in teenagers, with a reported frequency of 12.2%. In these young people, the frequency of a GFD was 2.9%, which is much higher than the percentage of known CD in the same population (1.26%). IBS symptoms were the most common clinical presentation in the self-reported NCWS teenagers. A greater awareness of the possible implications on the part of the subjects involved, and a more thorough medical approach to the study of the self-reported wheat-induced symptoms are warranted

## Figures and Tables

**Table 1:** Demographic characteristics of the subjects with self-reported Non-Celiac Wheat Sensitivity (NCWS) and the controls (number and percentage are given).

	Self-Reported NCWS (*n* = 68)	Controls (*n* = 480)	*p* Value
Mean Age (±SD)	17.8 ± 2.1	17.5 ± 2.2	N.S.
Sex (females)	46 (68%)	318 (66%)	N.S.
Father or mother graduated	29 (43%)	210 (44%)	N.S.
Parents unemployed (both)	6 (9%)	41 (8%)	N.S.
Engaged	32 (47%)	174 (36%)	0.07
Caucasian	68 (100%)	477 (99%)	N.S.

**Table 2:** Diseases previously diagnosed in subjects with self-reported NCWS and in controls. Number and percentage are given.

Diagnosed Diseases	NCWS (*n* = 68)	Controls (*n* = 480)	Odds Ratio	*p* Value
Anxiety	40 (58.8%)	261 (54%)	1.7 (0.98–2.8)	0.054
Depression	10 (14.7%)	38 (8%)	2.1 (0.96–4.3)	0.06
Bipolar disorder	5 (7.4%)	23 (5%)	1.6 (0.6–4.3)	N.S.
Thyroid diseases	1 (1.5%)	7 (1.7%)	1.02 (0.1–8.4)	N.S.
Diabetes mellitus	2 (2.9%)	6 (1.3%)	2.4 (0.5–12.3)	N.S.
Vitamin B12 deficiency	4 (5.9%)	14 (3%)	2.2 (0.7–2.8)	N.S.
Chronic fatigue syndrome	6 (9%)	13 (2.8%)	3.5 (1.3–9.6)	0.02
Fibromyalgia	0 (0%)	1 (0.2%)	0	N.S.
IBD	2 (2.9%)	4 (1%)	3.6 (0.6–20.4)	N.S.
Headache	3 (4.4%)	17 (3.6%)	1.3 (0.4–4.4)	N.S.
Food allergy	6 (9%)	10 (2%)	4.6 (1.6–13.1)	0.01
Lactose intolerance	8 (11.8%)	28 (5.8%)	2.2 (0.95–5)	0.06
GERD	9 (13.2%)	24 (5%)	2.9 (1.3.6.6)	0.02
Other	4 (5.9%)	19 (3.9%)	1.5 (0.5–4.6)	N.S.

## Abbreviations

NCWS	Non-celiac wheat sensitivity
GFD	Gluten-free diet
CD	Celiac disease
IBS	Irritable bowel syndrome
OR	Odds ratio
RR	Relative risk
SD	Standard deviation
MCA	Multiple correspondence analysis
